# New Hope for Pancreatic Ductal Adenocarcinoma Treatment Targeting Endoplasmic Reticulum Stress Response: A Systematic Review

**DOI:** 10.3390/ijms19092468

**Published:** 2018-08-21

**Authors:** Nuria Garcia-Carbonero, Weiyao Li, Marticela Cabeza-Morales, Javier Martinez-Useros, Jesus Garcia-Foncillas

**Affiliations:** Translational Oncology Division, OncoHealth Institute, Health Research Institute-University Hospital Fundación Jiménez Díaz-UAM, Avda Reyes Catolicos 2, 28040 Madrid, Spain; nuria.garciac@quironsalud.es (N.G.-C.); weiyao.li@quironsalud.es (W.L.); marticelacabezamorales@gmail.com (M.C.-M.)

**Keywords:** pancreatic ductal adenocarcinoma, ER stress, GRP78, ATF6α, IRE1α, PERK, ATF4, P38, UPR, dormancy

## Abstract

Pancreatic ductal adenocarcinoma (PDAC) is one of the most lethal types of tumours, and its incidence is rising worldwide. Although survival can be improved by surgical resection when these tumours are detected at an early stage, this cancer is usually asymptomatic, and disease only becomes apparent after metastasis. Several risk factors are associated with this disease, the most relevant being chronic pancreatitis, diabetes, tobacco and alcohol intake, cadmium, arsenic and lead exposure, certain infectious diseases, and the mutational status of some genes associated to a familial component. PDAC incidence has increased in recent decades, and there are few alternatives for chemotherapeutic treatment. Endoplasmic reticulum (ER) stress factors such as GRP78/BiP (78 kDa glucose-regulated protein), ATF6α (activating transcription factor 6 isoform α), IRE1α (inositol-requiring enzyme 1 isoform α), and PERK (protein kinase RNA-like endoplasmic reticulum kinase) activate the transcription of several genes involved in both survival and apoptosis. Some of these factors aid in inducing a non-proliferative state in cancer called dormancy. Modulation of endoplasmic reticulum stress could induce dormancy of tumour cells, thus prolonging patient survival. In this systematic review, we have compiled relevant results concerning those endoplasmic reticulum stress factors involved in PDAC, and we have analysed the mechanism of dormancy associated to endoplasmic reticulum stress and its potential use as a chemotherapeutic target against PDAC.

## 1. Introduction

Pancreatic ductal adenocarcinoma (PDAC) is the fourth leading cause of cancer death in both sexes, with an estimated 55,440 new cases of PDAC in United States in 2018 [1]. The incidence has increased worldwide, especially in northern European countries and Canada [2]. Moreover, some studies have estimated that pancreatic cancer would increase from the fourth to the second leading cause of cancer deaths in the United States by 2020 [3]. In Europe, PDAC-related mortality has increased from 75,439 deaths in 2009 to approximately 82,300 in 2014 [4]. This rise has been hypothesised to be related to the increase in the consumption of high-sugar or carbohydrate-rich foods [5]. The most crucial acquired risk factors for pancreatic cancer sorted by relevance are cigarette smoking (hazard ratio (HR) = 1.74), obesity (body mass index > 30; HR = 1.2–1.5), high alcohol consumption (HR = 1.1–1.5), and some infectious diseases that include *Helicobacter pylori* (HR = 1.5), *Hepatitis B virus* and *Human Immunodeficiency virus* [6,7,8]. Interestingly, other studies suggested that high consumption of cooking and table salt, and smoked food have been significantly linked with pancreatic cancer (*p* = 0.009, *p* = 0.0001, and *p* < 0.01 respectively) [9]. Other observational studies associated pancreatic cancer with cadmium, arsenic, and lead exposure [10]. Indeed, those European countries with the highest levels of arsenic (more than 10 µg/L [11]), that include Finland, Austria, Czech Republic, Slovakia, and Hungary are those with highest incidence of pancreatic cancer [12].

It is estimated that 5–10% of PDAC cases present a hereditary component [13]. *BRCA2* is the most commonly mutated gene in familial PDAC [14]. *PALB2* is considered to be another relevant PDAC susceptibility gene [15], and it has been described that PALB2 protein binds to BRCA2 protein and contributes to its function [16]. Germline alterations in ataxia telangiectasia mutated (*ATM*) gene have also been identified in hereditary PDAC patients [17]. Some familial syndromes also constitute risk factors for PDAC development [18]. Mutations in the *PRSS1* gene are responsible for hereditary pancreatitis, with a cumulative risk of developing PDAC of 40–55% [19]. Germline mutations in the *LKB1/STK11* tumour suppressor gene cause Peutz-Jeghers Syndrome (PJS). PJS patients have an 11–36% increased risk to develop several tumour types, including PDAC [20]. Familial adenomatous polyposis (FAP) is caused by inactivating mutations in *APC* and *MUTYH*, and it is considered a risk factor for hereditary PDAC [19]. Lynch Syndrome is caused by alterations in mismatch repair genes: *MLH1*, *MSH2*, *MSH6*, and *PMS2*, and it shows a cumulative risk of 4% for PDAC [21].

High throughput genetic profiling platforms are powerful tools for analysis of whole DNA, RNA, and non-coding RNA, especially microRNA and long non-coding RNA [22]. The mutational status of *BRCA2* is one of the most useful predictive biomarkers in clinical practice [23]. Concerning micro-RNAs, the overexpression of miR-21 was associated with a shorter disease-free survival in patients who received adjuvant gemcitabine after surgical resection [24], and miR-21 overexpression predicts resistance to 5-fluorouracil [25]. Furthermore, high miR-21 levels in plasma were associated with poor outcome in patients treated with induction chemotherapy followed by chemoradiotherapy [26].

PDAC diagnosis is usually late because the disease is often asymptomatic in early stages, and the first symptoms, such as abdominal pain and nausea, are usually managed in outpatient care. Also, diabetes has been associated with pancreatic cancer emergence, and it could be used as an early diagnosis biomarker (HR = 1.4–2.2) [27]. Complementary tests are performed when cholestasis, intestinal obstruction, or pancreatitis occur [28]. Prognosis is usually poor, with a 5-year survival of only 8% [29]. Survival can be improved when tumours are detected at early stages; indeed, it has been reported that 5-year survival rate is 50% when tumours are <2 cm [30], and close to 100% for tumours < 1 cm [31]. However, lesions < 1 cm or between 1 and 2 cm often go unnoticed on computed tomography (CT) or magnetic resonance imaging (MRI) scans.

Surgical resection is currently the best option to improve survival [32]. The mean life expectancy for pancreatic cancer is 1.4 years, reaching 3.5 years for surgically resected patients vs. 0.8 years for non-operated patients (*p* < 0.001) [33]. Resection criteria are described in the National Comprehensive Cancer Network (NCCN) guidelines [34]. After optimal resection (R0), the grade of cellular dysplasia usually determines the prognosis. However, other clinical variables such as pT, pN, pM, or the tumour stage may act as a prognostic tool in unresectable tumours [35].

Gemcitabine monotherapy was established as the first standard of care, due to the greater clinical benefit compared to 5-FU in alleviation of some symptoms [36]. However, its small survival improvement made it necessary to use gemcitabine in combination with platinum compounds [36]. Those compounds widely used in clinical practice are cisplatin, carboplatin, and oxaliplatin. They form DNA adducts, and especially crosslink DNA, which triggers the apoptosis cascade [37]. The expression of hENT1, which manages transport of gemcitabine and metabolically activate it, seems to be related to gemcitabine response. However, different antibodies used to determine hENT1 expression by immunohistochemistry demonstrated varying levels of predictivity of survival [38].

Dealing with locally advanced pancreatic cancer, a phase II trial suggested that a capecitabine-based regimen as induction chemotherapy is preferable in combination with radiation (50 Gy in 28 fractions) [39].

For metastasic PDAC, a combination chemotherapy regimen consisting of folinic acid, 5-FU, irinotecan and oxaliplatin (FOLFIRINOX) has demonstrated not only an increase in overall survival (*p* < 0.001), but also an increase in progression-free survival (*p* < 0.001), and in the objective response rate (*p* < 0.001) compared with gemcitabine as a first-line therapy. Nevertheless, the safety profile of FOLFIRINOX was less favourable than that of gemcitabine [40]. Other options of treatment for patients with metastatic disease and good performance status (0 or 1) include nab-paclitaxel. Paclitaxel destroys cancer cells by preventing the normal breakdown of microtubules during cell division, and albumin transports paclitaxel across the endothelial cell and concentrates it in the tumour. The combination of gemcitabine plus nab-paclitaxel extended the median overall survival (OS) of patients compared to gemcitabine alone, after 2 months [41].

Other chemotherapeutic approaches for those patients with presence of *BRCA2*, *PALB2*, *ATM*, or mismatch repair (*hMLH1* and *MSH2*) gene mutations are poly-ADP ribose polymerase (PARP) inhibitors, which subsequently cause an impair in DNA damage repair [42]. Remarkably, neoadjuvant iniparib plus gemcitabine induced a complete pathological response in a patient with recurrent PDAC harboring the *BRCA2* mutation [43]. However, in a phase I study of olaparib plus gemcitabine in patients with advanced solid tumours, which included 15 patients with PDAC, no differences were found in terms of efficacy [44].

Tumour cells can activate quiescent endothelial cells by overexpression of pro-angiogenic factors like vascular endothelial growth factor (VEGF) [45]. Nevertheless, anti-angiogenic therapy based on bevacizumab either in combination with gemcitabine or gemcitabine plus erlotinib has been unsuccessful for the treatment of metastasic PDAC [46].

Immunotherapy based on immune checkpoint inhibitors has risen to the forefront of therapeutic strategies against some solid tumors. Unfortunately, monotherapy with anti-CTLA4 monoclonal antibodies was ineffective in the treatment of locally advanced or metastatic PDAC [47].

More recently, the use of nanoliposomal irinotecan in combination with 5-fluorouracil/folinic acid has shown an improvement of OS over 5-fluorouracil/folinic acid alone (6.1 vs. 4.2 months), and it may constitute an active and tolerable second-line treatment option [48].

Nevertheless, patients could present acquired resistance to these chemotherapies due to the accumulation of secondary genomic alterations [49]. Thus, the identification of new therapeutic targets becomes a critical necessity for increase patients’ survival. PDAC is an aggressive tumour, and the similarity between 5- and 15-year survival rates suggests that the disease does not generate dormant cells [50]. Therefore, the therapeutic induction of dormancy may lengthen the survival of patients in future, and that is where targeting endoplasmic reticulum stress could come in.

Endoplasmic reticulum (ER) stress pathways have turned into a target in the effort to modulate the apoptosis of cancer cells. The objective of the present review is to increase the understanding of ER stress response factors as potential targets for PDAC treatment. Therapies based on drugs that could modulate these ER stress response proteins would open new routes for the management of PDAC patients.

A literature search of PubMed database (U.S. National Institutes of Health’s National Library of Medicine) was conducted to obtain research articles. Keywords such as ER stress response, unfolded protein response, PDAC treatment, GRP78, ATF6α, IRE1α, PERK, ATF4, and P38 were used in the computer-assisted literature search. After the articles were imported, the results from the studies were merged, with a total of 1220 articles retrieved. Of them, 1148 articles were excluded manually because they were unrelated to cancer (*n* = 141), unrelated to ER stress factors (*n* = 873), or had no treatment involvement (*n* = 62). Of the remaining 72 articles, 15 studies were excluded, and 57 met the inclusion criteria for showing both qualitative and quantitative data. Then, these 57 articles were critically appraised for the present ER stress review. The rest of the 49 citations were included for PDAC introduction. From each report, the following information was subsumed for the present systematic review: type of neoplasia studied, ER stress factor involved, description of such in vitro and/or in vivo approaches, treatment used and doses, statistical significance, molecular pathway involved, and most relevant conclusions.

All types of research articles that met the criteria of this review were included in the study regardless of whether the results were positive, negative, or null.

## 2. Endoplasmic Reticulum Stress Response in PDAC

**Figure 1:**
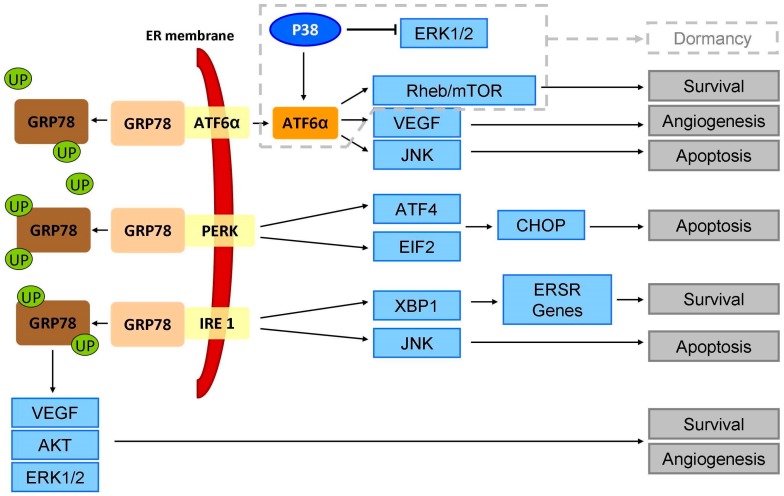
Consequences of endoplasmic reticulum stress response pathway. Unfolded protein accumulation and other stress conditions such as hypoglycemia and hypoxia cause the unbinding of GRP78 from ATF6α, PERK, and IRE1. These three factors activate different signalling pathways that lead to survival or to apoptosis. UP: Unfolded proteins; ER: Endoplasmic reticulum; ERSR: Endoplasmic reticulum stress response; VEGF: vascular endothelial growth factor; AKT: AKT serine/threonine kinase; ATF4: activating transcription factor 4; CHOP: CCAAT/enhancer binding protein (C/EBP)-homologous protein; JNK: c-Jun N-terminal kinase; EIF2: eukaryotic initiation factor 2; XBP1: transcription factor X-box 1.

Endoplasmic reticulum stress induces a signaling pathway that increases protein folding capability to maintain cell homeostasis. This response to stress is also called the unfolded protein response (UPR) or ER stress response (ERSR). Hypoglycemia and hypoxia also activate an ERSR, which physiologically generates mechanisms to prevent increased cell damage and avoid apoptosis. Nevertheless, if homeostasis cannot be restored and the cell is subjected to chronic stress, generalised apoptosis will be triggered [51]. ERSR involves three mechanisms: (i) transcription of the chaperone GRP78 to support protein folding; (ii) reduction of translation activity to minimise the quantity of unprocessed protein; and (iii) degradation of the accumulated unfolded proteins through the ubiquitin-proteasome pathway [52,53]. These three mechanisms are controlled by three transmembrane sensors in the ER, namely PERK, IRE1α, and ATF6α, which remain inactive while they are binding to the ER chaperone GRP78 [53]. PERK directly induces ATF4 and eIF2α, IRE1α activates XBP1, and ATF6α leads the activation of the JNK and Rheb/mTOR pathways (Figure 1).

Under ER stress conditions, unfolded proteins bind to GRP78. Then, GRP78 unbinds from these ER stress sensors and ERSR is triggered to prevent apoptosis and promote cell survival [54]. Nevertheless, some studies have shown that when ER functions cannot be restored by ERSR, long-term stress triggers apoptosis through CHOP activation [54,55]. Subsequently, ERSR promotes survival, and therefore it has been closely related to cancer development and progression [56].

GRP78 expression remains at low baseline levels in untransformed somatic cells; however, it is overexpressed in PDAC cells, and it has been associated with poor prognosis and tumour chemoresistance (Table 1 and Table 2) [57,58]. Tissue microarray-based immunohistochemistry with tumour and normal tissue from 180 PDAC patients showed that GRP78 expression was significantly increased in cancer cells compared to non-tumour tissue (*p* < 0.05). This highest expression of GRP78 was associated with a highest T-stage and poor overall survival (*p* < 0.05). In contrast, GRP78 silencing with small interfering RNAs (siRNAs) in PDAC cell lines decreases proliferation, invasion and migration [57]. In other study, GRP78 expression in PDAC cell lines was associated with invasive potential. Its overexpression in PDAC cell lines activated FAK and JNK that promoted invasion, and its downregulation inhibited the invasive potential [58]. Moreover, GRP78 plays an important role in chemoresistance. GRP78 downregulation with siRNAs in combination with chemotherapeutics increased cell death compared to treatment or individual silencing. GRP78 downregulation also decreased ABC transporter activity, sensitising the PDAC cells to several chemotherapeutic agents [59]. GRP78 overexpression contributed to tumour angiogenesis independently of vascular endothelial growth factor (VEGF) and it increased AKT phosphorylation and ERK1/2 activation [60]. In fact, GRP78 knockout mice showed a severe reduction of angiogenesis and tumour growth without any effect on normal tissue [61]. In other types of cancer, GRP78 has also been associated to tumour malignancy. Its suppression caused CD24 downregulation and enhanced human colorectal cancer cells sensitisation to oxaliplatin [62]. In addition, GRP78 inhibition with plumbagin contributed to apoptosis induction and sensitised cells to tamoxifen in breast cancer [63]. GRP78 targeting with antibodies exhibited a decrease in cell proliferation and tumour growth, apoptosis induction and improved efficacy of radiation therapy in human glioblastoma and non-small cell lung cancer [64].

Under ER stress, the activation of the PERK-eIF2α-ATF4-CHOP pathway triggers apoptosis and has been proposed as a new alternative to induce apoptosis by ER stress induction in pancreatic cell lines and mice models (Figure 1, Table 1) [55,65,66]. To counteract ER stress, PERK activates EIF2α by phosphorylation, which inhibits protein translation and reduces the influx of unfolded proteins into the endoplasmic reticulum; thus, PERK promotes pro-oncogenic capabilities [67]. It has been reported that PERK contributed to proliferation and angiogenesis in low-grade pancreatic neuroendocrine tumour models [68] (Table 1). Moreover, PERK enhanced invasion and metastasis in murine models of breast cancer, and it has been associated with poor prognosis of breast, lung, and gastric cancer [69] (Table 2).

Another factor activated by PERK is the transcription factor ATF4. It has been previously described that the role of ATF4 is related to tumour cell survival [70]. In haematological malignancies, ATF4 has been used as a target for treatment against cultured and primary cancer cells lines, and mice models [71,72] (Table 2). In colorectal cancer, overexpression of ATF4 has been related to oxaliplatin and 5-fluorouracil resistance [73], while ATF4 downregulation delayed tumour growth of endometrial cancer xenografts [74] (Table 2). ATF4 has also been found to be overexpressed in pancreatic neuroendocrine tumours [75]. Interestingly, ATF4 protein levels were increased after gemcitabine treatment in PDAC cell lines; therefore, ATF4 downregulation has been proposed as an additional mechanism to induce apoptosis in combination with gemcitabine [76].

IRE1 is another factor that is responsible for activating ERSR. In mammals there are two isoforms: IRE1α, which is ubiquitously expressed, and IRE1β, which is restricted to the respiratory and gastrointestinal tract [51]. Activation of IRE1α promotes the transcription factor X-box 1 (XBP1), which also induces the transcription of other ERSR genes (Figure 1) [51,54]. It has been reported that XBP1 is overexpressed in PDAC cell lines [77]. It has been suggested that the inhibition of IRE1α/XBP1 could be a target for future treatments for anticancer therapies [78]. Indeed, IRE1α induces the JNK pathway to conduct apoptosis through two mechanisms: nuclear translocation of c-Jun and activation, and overexpression of pro-apoptotic factors that include TNFα, FAS-L, and BAK2 [79]; and mitochondrial translocation of JNK, which cleaves BID, and activates the intrinsic apoptosis cascade (Figure 1). Moreover, JNK inhibits BCL2 family proteins, thus avoiding the anti-apoptotic properties of these proteins [80]. The use of IRE1α to induce apoptosis as a target for cancer management has been demonstrated in several pre-clinical studies. In acute myeloid leukemia, downregulation on IRE1α reduced cell viability, increased apoptosis ratio, and led to cell cycle arrest of tumour derived cell lines and primary cell lines [81]. Moreover, it has been reported that high levels of XBP1 suppressed an aggressive phenotype of breast cancer-derived stem cells [77] (Table 2). XBP1 offers an immunotherapeutic potential to induce CD3^+^CD8^+^ cytotoxic T lymphocytes against breast, colon, and pancreatic cancer [82]. In fact, the regulation of IRE1/XBP-1 increased drug sensitivity and decreased cell proliferation in breast cancer-derived cell lines and in mice models [83,84] (Table 2). However, to our knowledge, there are no studies of IRE1α that are directly related to pancreatic cancer.

ATF6 is another factor that is necessary to induce ERSR. ATF6 has two isoforms: α and β; however, it has been reported that ATF6β did not play a significant role in ERSR [85].

ATF6α is activated in the Golgi apparatus [86,87], is translocated to the nucleus, and stimulates transcription of survival genes to neutralise ER stress, avoiding apoptosis and promoting cell survival [85,88] (Figure 1). Moreover, ATF6α is considered an important promoter of VEGF to induce angiogenesis [89]. In pancreatic neuroendocrine tumours, downregulation of ATF6α is able to induce apoptosis through P38 activation [90] (Table 1). Our group has previously reported a two-protein signature based on high expression of ATF6α and low expression of active form of P38, which was associated with a high risk of recurrence in PDAC (HR = 3.256; 95% CI, 1.283–8.266; *p* = 0.013). Multivariate analysis revealed that a combination of high expression of ATF6α and a low expression of phospho-P38 was associated with a poor outcome for PDAC patients (HR = 2.705; *p* = 0.023), together with tumour grade (HR = 2.886; *p* = 0.029) [91] (Table 1). Concerning other types of tumours, a high expression of ATF6α has been involved in chemoresistance of leukemia-derived cell lines to imatinib [92], and it was necessary for the survival of melanoma-derived cell lines under ER stress conditions [93] (Table 2).

## 3. ERSR to Induce Dormancy in PDAC

One of the most crucial activated pathways in PDAC involves mitogen-activated protein kinases (MAPKs), which promote survival and induce proliferation [94]. However, some of the MAPK factors such as JNK and P38 negatively regulate cell cycle progression and induce cell death [95]. Expression of P38 limits cell-line growth by negative regulation of the cycle in the G1/S and G2/M transitions; pharmacologic inhibition of P38 was able to increase tumour cell proliferation in vitro and in xenografts. However, JNK inhibition was able to antagonise the effects of P38 inhibition [96]. Therefore, the role of P38 has been closely related to an apoptotic phenotype. In fact, it has been reported that high levels of P38 are correlated with longer survival in PDAC patients [91] (Table 1). In other tumour types like colorectal and breast cancer, the drug-specific activation of P38 selectively inhibited the self-renewal of cancer stem cells (CSC) through a reversal of stem cell markers (CD44 and CD133) and self-renewal factors (c-MYC and BMI-1). Thus, this study corroborated the recognised role of P38 as a tumour suppressor [97].

Interestingly, P38 is also involved in stem cell dormancy. This means that tumours release disseminating cells in a quiescent state that lodge in other organs, albeit in the initial stages, which will develop distant metastasis even after resection of the primary tumour [98,99]. This mechanism is used by cancer cells to become refractory to targeted or conventional therapies, and to prolong their survival by interactions with other different cell populations such as endothelial cells, immune cells, stromal cells, or fibroblasts [100].

P38 phosphorylation contributes to the nuclear translocation and activation of ATF6α in dormant cancer cells through the ATF6α/Rheb/mTOR pathway (Figure 1). The ERSR sensor ATF6α promotes survival through Rheb overexpression and activation of mTOR signalling pathways independently of AKT. Moreover, the interaction between ATF6α and mTOR signaling conferred resistance to doxorubicin and rapamycin, showing a mechanism of chemoresistance. Indeed, Schew and Aguirre-Ghiso reported that downregulation of ATF6α prolonged the survival of nude mice bearing dormant tumour cells [99].

Activation of P38 inhibits ERK1/2, which induces cell cycle inhibitors P27 and P21, keeping the cell in G0–G1 phase, which is crucial for maintaining a dormant phenotype [98]. Furthermore, P38 inhibits tumour transformation by oxidative stress [101] and it also cooperates with ERK1/2 to induce the quiescence of tumour cells [102].

ATF6α is expressed even in non-stress conditions, and it is necessary to maintain GRP78 expression. Indeed, ATF6α mRNA levels increased when ER stress was induced with ER stress-triggering drugs such as tunicamycin, thapsigargin, dithiothreitol (DTT), or high glucose concentration. ATF6α downregulation with small interfering RNA reduced GRP78 expression, and raised susceptibility to cell death. Thereby, β-cell apoptosis increased in ATF6α-depleted cells in either control or ER stress conditions [90] (Table 1).

There are currently no studies showing a direct relationship between ATF6α and dormancy of PDAC. In other tumour types, ATF6α overexpression has been proposed to be a target against cancer stem cells (CSC), due to the nuclear translocation of ATF6α under the effect of tunicamycin in CSC population [77] (Table 2). Recently, heparan hexasaccharide sulfate has been used with favourable results, selectively inhibiting CSC from PDAC and other solid tumours by activation of P38 [97] (Table 1 and Table 2). Although there are studies with promising results concerning PDAC, there is still a lack of evidence to justify the use of ERSR activation as a target for proliferative pancreatic cancer and stem cells. Nevertheless, the ERSR could be a potential pathway to increase patient survival.

Other tumours like prostate cancer, melanoma, or breast cancer show the highest 5-years survival rates (100, 93, or 90%, respectively) but survival decreases to ~20% at 15-years [50]. In these cases, dormant tumours cells could be present to generate metastasis years later. Translational research community wonders whether the “awakening” of dormant cells may or not facilitate their elimination with standard chemotherapies. However, the genetic heterogeneity of dormant cells suggests that treatments may not be as effective as was originally intended. In the case of PDAC, it has a 5-year survival rate of 6%, and survival is relatively unchanged at 10 and 15 years. This survival rate suggests that PDAC does not present dormancy. Then, dealing with PDAC, the implementation of a drug to induce dormancy would be the most effective strategy to longer survival rates and a delay in the appearance of metastases.

## 4. Conclusions

ERSR is a molecularly well-known mechanism that has recently been used for different treatment approaches in several types of cancer aimed both at proliferating and dormant tumour cells. Some of these strategies are directly related to ERSR factors, but others are indirectly associated with this molecular pathway, as in the case of P38. Regarding PDAC, few reliable data are available concerning ERSR. Additionally, current research is mainly focused on in vitro models, and even less frequently performed with in vivo models testing interference RNA-based technology. Therefore, until new specific targeted therapies appear to design future clinical trials, it will not be able to evaluate the real impact on patient’s survival. In fact, clinical trials to perform the validation of these pre-clinical results are generally limited, due to the low incidence of this kind of tumour.

It has been suggested that there are several ways to trigger apoptosis and to induce response to anti-tumour drugs. Those mechanisms include several pathways. One of them is the induction of ER stress by the inhibition of GRP78/BiP unbinding from transmembrane sensors PERK, ATF6α, and IRE1α. Out of these, GRP78 is the most widely studied protein that is related to the ER stress response.

The knowledge of ERSR is leading to new studies, which allows the generation of potential targeted therapies for those cancers with limited treatment options, such as PDAC. Forcing the cell into a chronic state of ER stress or restraining its response via ATF6α/JNK, IRE1α/JNK, or PERK/CHOP pathways could be a promising approach to achieve cell cycle arrest, apoptosis, and chemosensitivity, and to reduce invasive phenotype and angiogenesis.

On the other hand, the induction of dormancy is not a phenomenon that is widely studied in cancer, let alone in PDAC. Therefore, further studies are needed to be able to induce an effective and sustained cell dormancy. From our point of view, modulation of dormancy in disseminated proliferative tumour cells with adjuvant targeted therapies after R0 resection could be a potential successful treatment option to delay the emergence of distant metastases, and to prolong PDAC patients’ survival.

## Figures and Tables

**Table 1:** Pre-clinical studies with ER stress response factors against pancreatic ductal adenocarcinoma.

Factor	Material	Treatment (Concentration)	ER Stress Factors Involved	*p*	Brief	Ref
ATF6	45 patient samples (stage II and III)	none	ATF6α/P38	=0.013	ATF6α high expression and P38 low expression associates with poor outcome	[91]
GRP78	180 patient samples (stage I, II and III)	none	GRP78/CDK4/CDK6/STAT3/JAK2/RhoA	<0.05	GRP78 overexpression associates with poor outcome	[57]
	Cell lines (MIA PaCa-2, S2-VP10, SU.86.86)	Gemcitabine (400 nm), paclitaxel (50 nm), 5-FU (5 μm), GRP78 small interfering RNA (siRNA)	GRP78/ABC transporters	<0.001	GRP78 downregulation diminishes chemoresistance and increases apoptosis when combined with chemotherapeutics	[59]
	Cell lines (Panc-1, CFPAC1, MIA-PaCa-2, Panc2.03, Panc02). Mouse samples (Panc-1 xenograft; Panc02 orthotopic implantation)	Cell lines: Erastin (2.5–40 μm), GRP78 short hairpin RNA (shRNA), ATF4 shRNA. Mice models: gemcitabine (20 mg/kg), sulfasalazine (SAS; 100 mg/kg/i.p.), gemcitabine + sulfasalazine, or gemcitabine + sulfasalazine + liproxstatin-1 (10 mg/kg/i.p)	ATF4/GRP78/GPX4	0.05	GRP78 decreases ferroptosis and limits gemcitabine sensitivity both in vitro and in vivo	[103]
	Mice models (Pdx1-Cre; Kras^G12D/+^; p53^f/+^)	GRP78 genetically modified mice	GRP78	<0.01	GRP78 haploinsufficiency suppresses acinar-to-ductal metaplasia and tumorigenesis	[104]
	Cell lines (BxPC-3, Panc-1, Capan-1, Capan-2, CFPAC-1, HPAF-II, AsPC1). Mouse models (xenografts via subcutaneous injection)	ONC212 (cell lines: 20 μm; mice: 50 mg/kg)	GRP78/IGF1-R	<0.05	ONC212 shows an anti-proliferative effect and induces apoptosis, reducing tumour growth by inducing UPR	[105]
PERK	Cell lines (BxPC3). Mouse models (human tumour xenografts)	Cells lines: GSK2656157 (1 μmol/L), tunicamycin (5 μg/mL) and thapsigargin (1 μmol/L). Mice: GSK2656157 (50 or 150 mg/kg, orally)	PERK/EIF2α/ATF4/CHOP	<0.05	PERK inhibition as a potential anti-tumour and anti-angiogenic approach	[65]
	Cell lines (Panc-1, PK1, KLM1)	Avarol (40 μm)	GRP78/PERK/EIF2α/CHOP	<0.01	Avarol induces apoptosis via CHOP	[55]
P38	35 patient samples (stage I, II, III and IV). Cell lines (Panc5.04, Panc2.5, HPNE-E6/E7, HPDE). Mouse models (xenografts)	Cells lines: 5, 10, or 20 μmol/L. In vivo: intraperitoneal injection of SB202190 (2.5 mg/kg/day), and SP600125 (40 mg/kg/day)	P38/JNK	=0.041	Active P38 contributes to better outcome	[96]
	Cell lines (Panc-1), and derived cancer stem cells	Heparan sulfate hexasaccharide (100 μm)	P38/TCF4	<0.005	P38 activation inhibits cancer stem cells self-renewal inhibition	[97]
ATF4	Cell lines (AsPC-1, Panc-1)	ISRIB (250 nm), Gemcitabine (1 μm), ATF4 siRNA	EIF2/ATF4/CHOP	<0.01	ATF4 downregulation induces apoptosis in combination of gemcitabine	[76]
	Cell lines (Panc-1, HepG2, MIA PaCa-2)	Acriflavine (2.5 μm)	PERK/eIF2α/ATF4	<0.001	Acriflavine restores drug sensitivity by blocking UPR and EMT	[106]
PERK/ATF6	Mouse models (BxPC-3 xenografts)	Tanshinone IIA (0, 30 or 90 mg/kg)	PERK/ATF6/caspase-12/IRE1α/elF2α/p-JNK/CHOP/caspase-3	<0.001	Tan-IIA promotes apoptosis by induction of ER stress	[66]

ATF6: activating transcription factor 6; GRP78: 78 kDa glucose-regulated protein; PERK: protein kinase RNA-like endoplasmic reticulum kinase; ATF4: activating ranscription factor 4; UPR: unfolded protein response; EMT: epithelial-mesenchymal transition; ER: endoplasmic reticulum; Ref: references.

**Table 2:** Pre-clinical studies with ER stress response factors in other solid tumours and haematological malignancies.

Factor	Disease	Material	Treatment (Concentration)	ER Stress Factors Involved	*p*	Brief	Ref
ATF4	Pancreatic neuroendocrine tumour	45 patient samples	-	GRP78/ATF4/CHOP	<0.05	ATF4 is overexpressed in pancreatic neuroendocrine tumours	[75]
	MCL and AML	MCL cell lines (Z-138, JVM-2, MINO, and JeKo-1). AML cell lines (OCI-AML3, MOLM-13, HL-60, and THP-1). Primary cells. Mouse models (via tail vein injection)	ONC201 (5 μm), rapamycin (10 nm), or tunicamycin (1 μm)	ATF4/mTORC1	<0.0001	ONC201 induces apoptosis independent of TP53 mutation status and causes changes in gene expression similarly by UPR. ONC201 induces ATF4 and inhibits mTORC1	[71]
ATF4-ATF3-CHOP	TLL	TLL cell lines (Jurkat, Molt4) and the T-cell hybridoma cell line (DO11.10)	Farnesol (75 μm)	ATF4/ATF3/CHOP/PERK-eIF2α	<0.01	Farnesol induces apoptosis in leukemic cells by induction of the PERK-eIF2α-ATF3/4 cascade	[72]
ATF4	CRC	CRC cell lines (HCT116 and LoVo)	Glucose deprivation (1,5 mmol/L glucose)	GRP78/PERK/ATF4	<0.001	Glucose deprivation protects cells from oxaliplatin- and 5-fluorouracil-induced apoptosis, and induces the expression of ATF4. Depletion of ATF4 can induce apoptosis and drug re-sensitisation.	[73]
ATF6	Insulinoma	Cell lines isolated from rat and mouse pancreas (INS-1 832/13)	Tunicamycin (0.1 µg/m), thapsigargin (0.1 µg/m), staurosporin, SB239063 (50 µm), and SP600125 (50 µm), ATF6α siRNA	GRP78/ATF6α	<0.05	ATF6α knockdown activates JNK and P38 to induce apoptosis in insulinoma cells and primary islets	[90]
	OC and CML	OC cell lines (HeLa), and CML cell lines (K562 and LAMA)	Dithiothreitol (1 mm), thapsigargin (500 nm), azetidine-2-carboxylic acid (10 mm), and tunicamycin (5 μg/mL)	PDIA5/ATF6/BiP	<0.01	PDIA5/ATF6α axis modulates sensitivity of leukemia cells to imatinib	[92]
IRE1α	AML	AML cell lines (NB4, U937, K-562, TF-1, HL-60, PL-21, and THP-1). Primary samples and murine hematopoietic cells	2-hydroxy-1-naphthaldehyde (25 μm), STF-083010 (50 μm), and toyocamycin (500 nm)	IRE1α/XBP1	<0.01	Inhibition of IRE1α decreases cell viability and induces apoptosis and G1 cell cycle arrest	[81]
IRE1/ATF6	Melanoma	Melanoma cell lines (Mel-RM, Mel-RMu, Mel-CV, and MM200)	siRNA and shRNA of IRE1α and ATF6	IRE1α/ATF6	<0.05	IRE1α and ATF6 are critical for survival of melanoma cells undergoing ER stress	[93]
IRE1/XBP1	BC	BC cell lines (MDA-MB-231 and MCF-7)	Thapsigargin (250 nm) or bortezomib (100 nm)	IRE1/XBP-1	<0.05	Estrogen receptor β sensitises BC cells to thapsigargin and to bortezomib by regulating the IRE1/XBP-1 pathway	[83]
	BC	BC cell lines (SUM159, BT549, and MDA-MB-231), PDX models, and genetically engineered mouse models	Small molecule inhibitor 8866 (300 mg/kg oral daily)	IRE1/XBP1 pathway and MYC	<0.001	Silencing of XBP1 selectively blocks the growth of MYC-hyperactivated cells. Pharmacological inhibition of IRE1 selectively restrained MYC-overexpressing tumour growth in vivo in a cohort of preclinical patient-derived xenograft models and genetically engineered mouse models	[84]
XBP-1	BC	CSC derived from MCF7 cell line (CD44+/CD24-)	Tunicamycin (2 μg/mL)	XBP-1/ATF6/CHOP	<0.001	Tunicamycin inhibited invasion, increased cell death, suppressed proliferation, and reduced migration in a CD44+/CD24- and CD44+/CD24- rich MCF7 cell culture by an increased level of spliced XBP-1, ATF6 nuclear translocation and CHOP protein expression	[77]
P38α/β	CRC and BC	CRC cell lines (HT29, HCT116, and LS174T) and BC cell lines (MDA-MB-231)	Heparan sulfate hexasaccharide (100 µm)	TCF4	<0.005	Heparan sulfate hexasaccharide selectively inhibits CSC self-renewal and induces apoptosis in colorectal and breast CSCs	[97]
GRP78	CRC	CRC cell lines (HT29, HT8, SW480 and colo205)	Oxaliplatin (5 µm) and vomitoxin (1 µg/mL)	GRP78/CD24	<0.001	Suppression of GRP78 sensitises human colorectal cancer cells to oxaliplatin by downregulation of CD24	[62]
	BC	BC cell lines (MCF-7 and T47D)	Plumbagin (from 0.5 to 5 μm) and Tamoxifen (1 or 5 μm)	GRP78/BIK	< 0.05	Plumbagin inhibits GRP78 activity, and increases Bik expression and apoptosis induction, which contributes to the sensitisation of BC cells to tamoxifen	[63]
	NSCLC and GB	NSCLC cell lines (A549, and H460), and GB cell lines (D54 and U251). Mouse xenografts.	Anti-GRP78 antibody (1 μg/mL)	GRP78 and PI3K/AKT/mTOR signaling	<0.0001	Anti-GRP78 attenuates cell proliferation, enhances radiation therapy, induces apoptosis, and delays tumour growth in mouse xenograft through the suppression of Akt/mTOR signaling	[64]
PERK	Insulinoma	Mice samples (insulinoma generated by SV40 Large T-Antigen)	ISRIB (250 nm), Gemcitabine (1 μm), ATF4 siRNA	PERK	<0.000005	PERK promotes tumour proliferation and angiogenesis	[68]
	Breast, lung and gastric cancer	BC cell lines (MCF7, T47D, BT474, BT549, ZR-75–30, Hs578T, MDA-MB-157, and MDA-MB-231). Orthotopic injection into a mammary pad on NOD/SCID mice. Breast, lung and gastric cancer patient samples.	AEBSF (1 mg)	PERK/CREB3L1/ATF4	< 0.01	PERK signalling drives invasion and metastasis of breast cancer cell lines through CREB3L1, and associates with a poor outcome in breast, lung, and gastric cancer patients	[69]

AML: acute myeloid leukemia; BC: Breast cancer; CML: chronic myeloid leukemia; CSC: Cancer stem cell; CRC: Colorectal cancer; EC: endometrial cancer; ER: endoplasmic reticulum; GB: Glioblastoma; NOD/SCID: non-obese diabetic/severe combined immunodeficiency; NSCLC: non-small cell lung cancer; MCL: mantle cell lymphoma; OC: Ovarian Cancer; PDX: patient-derived xenograft; TLL: T lymphoblastic leukemia; UPR: unfolded protein response; Ref: references.

## Abbreviations

The following abbreviations are used in this manuscript:

ATF6α	activating transcription factor 6 isoform α
AKT	AKT serine/threonine kinase
ATM	Ataxia telangiectasia mutated
CHOP	CCAAT/enhancer binding protein (C/EBP)-homologous protein
CI	confident interval
CSC	cancer stem cells
GRP78	78 kDa glucose-regulated protein
CT	computed tomography
eIF2α	eukaryotic initiation factor 2 isoform α
EMT	epithelial-mesenchymal transition
ER	endoplasmic reticulum
ERK	extracellular signal-regulated kinase
ERSR	endoplasmic reticulum stress response
FAP	Familial adenomatous polyposis
HR	hazard ratio
IRE1	inositol-requiring enzyme 1
JNK	c-Jun N-terminal kinase
MAPKs	mitogen-activated protein kinases
MRI	magnetic resonance imaging
NCCN	National Comprehensive Cancer Network
OS	overall survival
PARP	poly ADP ribose polymerase
PDAC	pancreatic ductal adenocarcinoma
PERK	protein kinase RNA-like endoplasmic reticulum kinase
PJS	Peutz-Jeghers Syndrome
UP	unfolded protein
UPR	unfolded protein response
VEGF	vascular endothelial growth factor
XBP1	transcription factor X-box 1
